# Primary Gastric Leiomyosarcoma: A Rare Case

**DOI:** 10.7759/cureus.49510

**Published:** 2023-11-27

**Authors:** Tengfei Wang, Riyam Zreik, Bing Leng

**Affiliations:** 1 Pathology, Baylor Scott and White Health, Temple, USA

**Keywords:** helicobacter pylori, low-grade, gastrointestinal stromal tumor (gist), leiomyosarcoma, stomach

## Abstract

Gastric leiomyosarcoma is extremely rare. In this paper, we present a case of primary gastric leiomyosarcoma located in the fundus/cardia region. The tumoral spindle cells show diffusely moderate nuclear atypia, with focally marked atypia and rare mitotic figures. Additionally, the tumoral cells exhibit positive immunoreactivity to smooth muscle actin and desmin while testing negative for CD117 (c-kit). The tumor was successfully resected through a laparoscopic partial gastrectomy, and the patient experienced a full recovery. There has been no recurrence or metastatic tumor detection during the seven-year follow-up period. Furthermore, we conducted a literature review on primary gastric leiomyosarcoma.

## Introduction

Leiomyosarcoma, a tumor originating from smooth muscle, represents an important differential diagnosis for gastrointestinal stromal tumors (GISTs). Prior to the 2000s, there were more documented cases of gastrointestinal (GI) tract leiomyosarcomas in the literature, largely due to the misclassification of many GISTs as leiomyosarcomas [1]. In the late 1990s, definitive immunohistochemical methods for distinguishing GIST from leiomyosarcoma were described. Diagnosis of primary leiomyosarcoma now relies on a combination of histomorphologic features, positive immunoreactivity to smooth muscle antigens, and non-responsiveness to GIST immunomarkers, namely CD117 (c-kit), CD34, and the discovery of gastrointestinal stromal tumor 1 (DOG1) [2]. Currently, primary leiomyosarcomas of the GI tract are rare, with leiomyosarcoma in the stomach being an exceptionally rare tumor among these malignancies. Unfortunately, reliable statistics regarding the demographic and clinicopathological characteristics of gastric leiomyosarcoma remain scarce [3]. In this article, we present a case of primary gastric leiomyosarcoma and provide a review of the relevant literature.

## Case presentation

A 66-year-old obese female presented with an eight-month history of gastroesophageal reflux symptoms and watery diarrhea. Her past medical history included colon cancer, gastric polyps, melanoma, and a right acoustic neuroma. She had no history of smoking or drinking. Notably, her first-degree relatives had various cancers, i.e., colon cancer, melanoma, and thyroid cancer. She had been managing her diarrhea symptoms symptomatically and was not taking proton pump inhibitors. Blood tests and physical examinations showed no remarkable findings.

**Figure 1:**
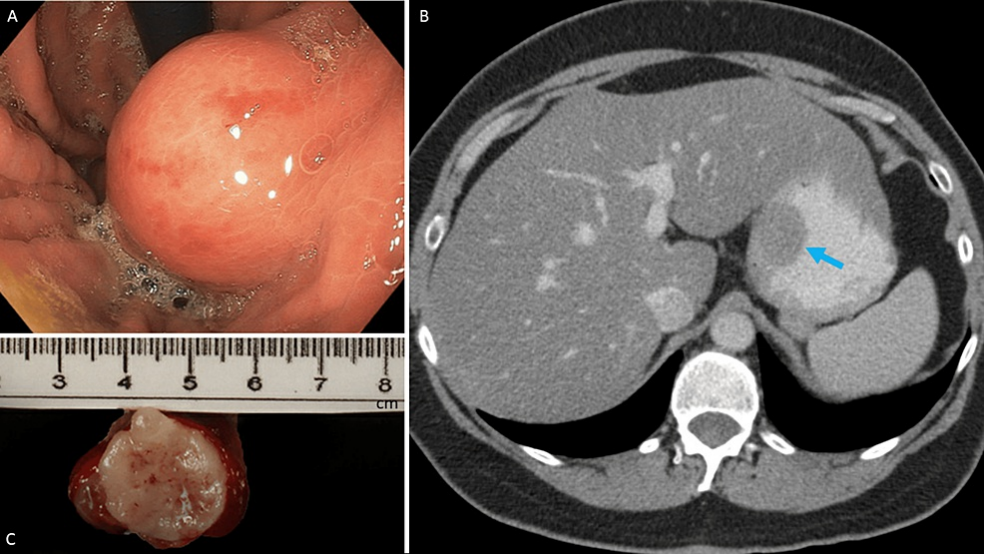
Mass in the gastric fundus/cardia region. (A) A submucosal lesion was revealed in EGD; (B) CT scan indicated an ovoid intraluminal mass. blue arrow: mass, approximately 3.0 cm × 2.3 cm; (C) the gross appearance of gastric leiomyosarcoma.

Biopsies of the duodenum and colon yielded insignificant results. An esophagogastroduodenoscopy (EGD) identified a submucosal lesion larger than 2 cm in the fundus/cardia region (Figure 1A), close to the esophagogastric junction. An endoscopic ultrasound (EUS) noted the lesion with a solid, homogeneous appearance consistent with a GIST. Given the tumor size and clinical impression of GIST, surgery was recommended. The lesion was marked with tattoo ink for the surgeons. The tumor biopsy, or fine needle aspiration (FNA), was not taken. A pre-operative abdominal computed tomography (CT) scan (Figure 1B) showed an approximately 3.0 cm × 2.3 cm ovoid intraluminal mass within the stomach near the gastric fundus, concerning neoplasms such as GIST tumors, with no evidence of metastatic disease.

Later, the patient underwent a laparoscopic partial gastrectomy to excise the tumor. The patient was in a supine position on the operating table. An incision was made lateral to the umbilicus, with two additional trocars on the patient's left side and one additional trocar on the patient's right side. A hand port was made in the patient's left upper quadrant to remove the surgical specimen.

**Figure 2:**
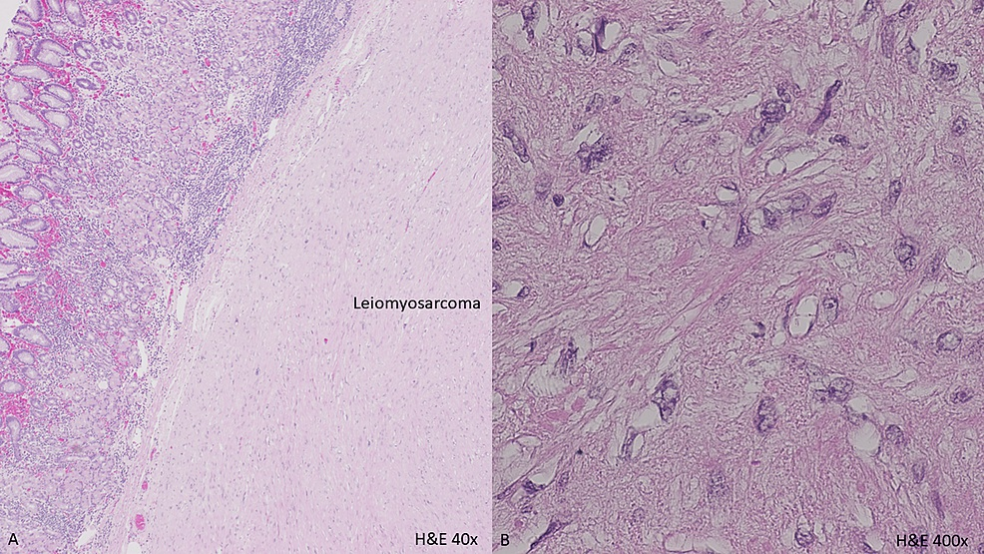
Microscopic appearance of gastric leiomyosarcoma. Intersecting fascicles of spindle tumoral cells (A, H&E, 40×) with abundant eosinophilic cytoplasm and cytologic atypia (B, H&E, 400×).

**Figure 3:**
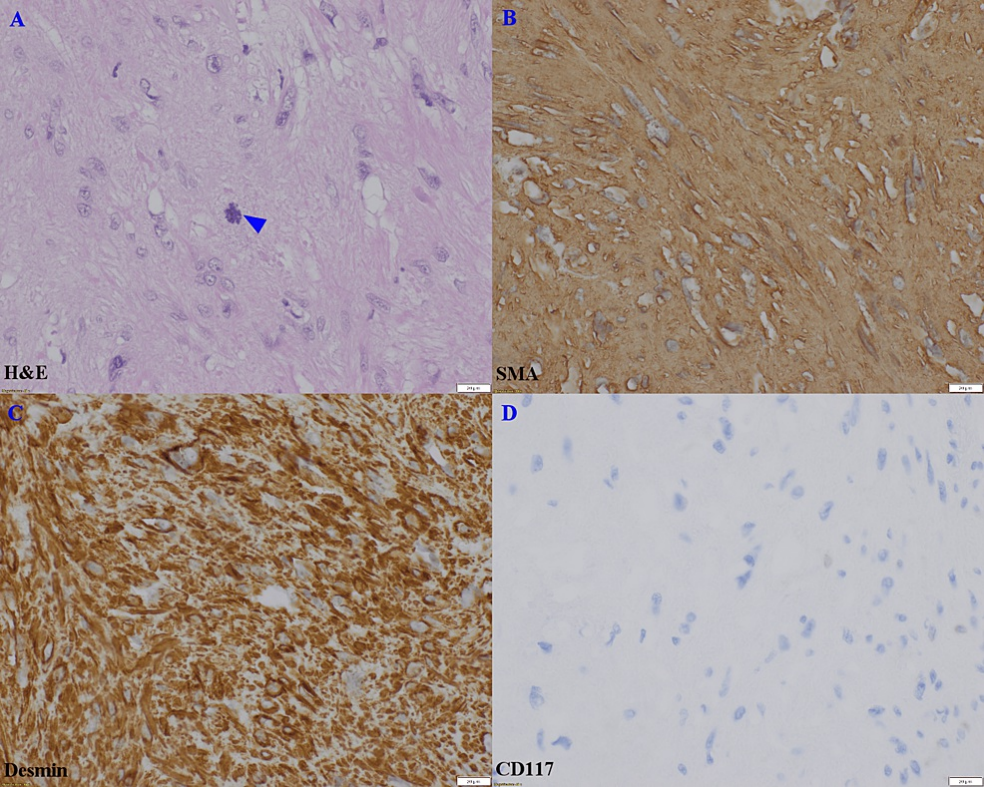
Histology and immunostains of the primary gastric leiomyosarcoma. A mitotic figure (A, H&E, arrowhead), tumor cells were positive for SMA (B) and desmin (C) but negative for CD117 (D). Scale bar: 20 µm.

The specimen consisted of a 5.3 cm × 3.0 cm × 2.4 cm portion of gastric mucosa with an underlying 3.8 cm × 2.0 cm × 1.8 cm tumor and a 6.5 cm × 1.0 cm × 0.8 cm portion of greater curvature stomach. The tumor appeared as a well-circumscribed, tan-white, firm submucosal nodule exhibiting a lobulated cutting surface and a few hemorrhagic foci (Figure 1C). Microscopically, the tumor originated from the muscularis propria of the stomach, consisting of intersecting fascicles of spindle tumoral cells with abundant eosinophilic cytoplasm (Figure 2). The nuclei displayed diffusely moderate and focally marked cytologic atypia, with rare mitotic figures not exceeding two per 10 high-power fields (HPF) (Figure 3A). Occasionally, atypical mitosis is noted. Some areas showed myxoid change, but no necrosis was identified. The tumor cells were positive for smooth muscle actin (SMA) and desmin while being negative for CD117 (Figure 3B-3D). The resection margin was narrowly free of tumors, and there was no evidence of lymphovascular invasion. The diagnosis, upon consultation, was low-grade gastric leiomyosarcoma (Fédération Nationale des Centres de Lutte Contre le Cancer (FNCLCC) Grade I of III). Additionally, the stomach showed mild activity and was positive for Helicobacter pylori.

The patient made a full recovery without any complications. Follow-up consisted of an annual abdominal CT scan for seven years, with no recurrence or metastasis detected.

## Discussion

We present a case of primary gastric leiomyosarcoma in an elderly female patient who also had a Helicobacter infection. The tumor was located in the gastric fundus and arose from the muscularis propria. This tumor was classified as low-grade (FNCLCC Grade I of III). Subsequent annual follow-ups, following excision via laparoscopy, revealed no recurrence or metastasis.

The etiology of stomach leiomyosarcoma remains unclear. However, there is a possibility that Helicobacter pylori plays a role in the development of gastric leiomyosarcoma, given its involvement in the pathogenesis of gastric mucosa-associated lymphoid tissue lymphoma and carcinoma [4]. Although a lack of KIT and PDGFRA mutations was reported in gastric leiomyosarcoma [2], further analysis is needed to identify the relevant genetic mutations. For our patient, a Helicobacter pylori infection was identified. She also had a history of multiple malignancies, i.e., colon cancer, melanoma, and acoustic neuroma, indicating potential underlying genetic mutations.

Gastric leiomyosarcoma commonly originates from the muscularis propria or muscularis mucosa layer in various locations of the stomach, including the body, fundus, antrum, cardia, and pylorus [5,6]. Clinical signs and symptoms often include abdominal pain, melena, weight loss, and the presence of an abdominal mass [7,8].

A recent comprehensive review [5] identified 19 documented cases of primary gastric leiomyosarcomas worldwide with reliable data. Among these cases, more men were affected than women (11 males vs. 8 females), with ages ranging from 16 to 74 years old. The recorded tumor sizes varied from 1 to 18 cm. Three of 19 (15.8%) patients died of this disease.

Endoscopic ultrasonography [9], contrast-enhanced CT scans [10], and MRI [11] are all effective in detecting gastric leiomyosarcomas. Currently, the preferred treatment method is surgical resection [1]. Laparoscopic gastrectomy [5,12] and endoscopic submucosal dissection [13] have been used for the removal of gastric leiomyosarcoma. In cases where gastric leiomyosarcoma has spread to other organs, treatment options may include chemotherapy, chemoembolization, and high-intensity targeted ultrasound therapy [1,14].

The FNCLCC grading system and the College of American Pathologists protocol for soft tissue tumors are commonly used to grade and stage gastric leiomyosarcoma, respectively [8]. The prognosis for patients with stomach leiomyosarcomas remains poorly understood. Potential prognostic factors for gastric leiomyosarcomas include histopathological grade and type, synchronous metastasis, and parietal gastric infiltration [10,15].

A recent study [16] investigated 407 cases of intramural smooth muscle tumors (SMTs) in the GI tract, confirmed through immunohistochemical staining. This extensive study involved data collected from 31 institutions across the United States, including 180 cases in the stomach with an average gastric tumor size of 5.7 cm. However, it is worth noting that detailed clinicopathological data regarding gastric SMTs was not available. A significant finding in this study was that SMTs outside of the esophagus - those in the stomach, small intestine, and colorectum - measuring larger than 10 cm and exhibiting ≥3 mitotic figures per 5 mm² tend to demonstrate more aggressive behavior and progression. Intriguingly, the authors also proposed classifying tumors into leiomyoma, leiomyosarcoma, and atypical smooth muscle tumors based on tumor size and mitotic rate. It is important to note that this proposal has not yet gained widespread acceptance.

Our experience highlights the diagnostic challenges associated with gastric leiomyosarcoma when it is low-grade. In our case, endoscopic ultrasonography proved valuable in delineating the tumor's boundaries. Furthermore, as demonstrated in our case, laparoscopic excision can be considered as a treatment approach for stomach leiomyosarcoma.

## Conclusions

Primary gastric leiomyosarcoma is an extremely rare disease. The diagnosis can be challenging, especially for low-grade leiomyosarcoma. Information regarding the clinicopathological correlation of the tumor is limited. Our case shows that the outcome of low-grade, small-sized gastric leiomyosarcoma can be optimistic.
